# Retinal Pigment Epithelial Cells Express a Functional Receptor for Glucagon-Like Peptide-1 (GLP-1)

**DOI:** 10.1155/2013/975032

**Published:** 2013-11-06

**Authors:** Alessandra Puddu, Roberta Sanguineti, Fabrizio Montecucco, Giorgio L. Viviani

**Affiliations:** ^1^Department of Internal Medicine, University of Genoa, IRCCS Azienda Ospedaliera Universitaria San Martino–IST Istituto Nazionale per la Ricerca sul Cancro, 6 Viale Benedetto XV, 16132 Genoa, Italy; ^2^First Clinic of Internal Medicine, Department of Internal Medicine, University of Genoa, IRCCS Azienda Ospedaliera Universitaria San Martino–IST Istituto Nazionale per la Ricerca sul Cancro, 6 Viale Benedetto XV, 16132 Genoa, Italy; ^3^Division of Cardiology, Geneva University Hospitals, Faculty of Medicine, Foundation for Medical Researches, 64 Avenue Roseraie, 1211 Geneva, Switzerland

## Abstract

Glucagon-like peptide-1 (GLP-1) is a gut-derived incretin hormone that has been shown to improve glucose homeostasis in type 2 diabetes. The biological effects of GLP-1 are mediated by its specific receptor GLP-1R that is expressed in a wide range of tissues, where it is responsible of the extra-pancreatic effects of GLP-1. Since the retinal pigment epithelium (RPE), that forms the outer retinal barrier, has a key role in protecting from diabetic retinopathy (DR), we investigated the potential expression and function of GLP-1R in a RPE cell line. ARPE-19 cells were cultured in DMEM/F12 supplemented with 10% FBS. The expression of GLP-1R was evaluated at both mRNA and protein levels. Then, the activation postreceptor intracellular signal transduction pathways (extracellular signal-regulated kinases 1 and 2 [ERK1/2] and protein kinase B [PKB]) were assessed by western blot in normal cells or silenced for GLP-1R in the presence or absence of 10 nmol/L GLP-1. The potential connections between intracellular signalling pathways triggered by GLP-1 stimulation were performed before incubating cells with kinase pharmacological inhibitors of mitogen-activated protein kinase (MEK)1/2, phosphatydilinositol-3kinase (PI3K), or epidermal growth factor receptor (EGFR). The results showed that GLP1R is expressed at both mRNA and protein level in ARPE-19 cells. Stimulation with GLP-1 strongly activated PKB and ERK1/2 phosphorylation till 40 min of exposure. GLP-1-mediated activation of both kinases was dependent on the upstream activation of PI3K and EGFR. Finally, treatment with GLP-1 did not affect the spontaneous release of VEGF-A from ARPE-19 cells. In conclusion, this paper showed that the presence of functional GLP-1R is expressed in RPE cells. These data might represent the rationale to further investigate the potential direct beneficial effects of GLP-1 treatment against DR.

## 1. Introduction

Glucagon-like peptide-1 (GLP-1) is a potent glucoincretin hormone released from intestinal L-cells in response to nutrient ingestion [1, 2]. This molecule was shown to improve glucose homeostasis not only potentiating glucose-dependent insulin release (main action), but also suppressing glucagon secretion and appetite and increasing *β*-cell mass. More recently, GLP-1 was shown to upregulate proinsulin gene transcription and insulin production also in pancreatic beta-cells not exposed to glucose, but stimulated with other mediators of injury [3–7]. These protective effects were shown to be mediated by GLP-1 binding to its cognate high-affinity receptor (GLP-1R), a glycosylated protein member of the G-protein-coupled receptor superfamily [8]. GLP-1 binding to GLP-1R activates various intracellular signalling pathways that have been mainly identified in pancreatic beta-cells [9–11]. However, recent evidence showed that GLP-1R is expressed also in peripheral tissues, including the central and peripheral nervous systems, heart, kidney, lung, and gastrointestinal tract [12], suggesting that GLP-1 might be directly protective not only on glucose homeostasis, but also on diabetes complication in peripheral organs. Recently, treatment with exedin-4 (a long-acting agonist of GLP-1R) was shown to reduce development of diabetic retinopathy ([DR], one of the commonest complications of diabetes) in animal models of diabetes, [13–15]. DR is considered as a neurodegenerative disease of the eye, characterized by loss of neuronal cells with a progressive alteration of retinal microvasculature, increased vascular permeability, and pathologic intraocular neovascularization [16–18]. The pathogenesis of DR is associated with changes in the activities of retinal pigment epithelium (RPE), a monolayer of highly specialized cells located between the retinal photoreceptors and the choroidal vasculature [19, 20]. Since RPE cells play a central role in retinal homeostasis by forming the outer retinal barrier and supporting the function of photoreceptors, in the present study, we investigated whether the GLP-1/GLP1R axis was expressed and functional in RPE cells.

## 2. Methods

### 2.1. Cell Culture and Experimental Conditions

The human cell line ARPE-19 (American Type Culture Collection, Manassas, VA, USA) was grown in a 1-to-1 ratio of DMEM/F12 (Cambrex Bio Science, Walkersville, MD, USA) supplemented with 10% FBS, 2 mmol/L-glutamine (Sigma-Aldrich, Milan, Italy), and antibiotics (100 U/mL penicillin G and 100 *μ*g/mL streptomycin sulphate) (Sigma-Aldrich, Milan, Italy). Cells were maintained at 37°C in a humidified 5% CO_2_ air incubator. The cell medium was replaced every 2 days. Cells were grown to confluence, removed with trypsin-EDTA (Sigma-Aldrich, Milan, Italy), and then seeded in multiwell dishes for the stimulations. On the basis of recent publications [3, 4], the dose of 10 nmol/L for GLP-1 (AnaSpec, Fremont, CA, USA) was selected and used in this study. This concentration corresponds to a maximal effective concentration that is broadly used in the literature and to plasma concentration of the incretin following a high-carbohydrate meal [8, 21, 22].

### 2.2. Cell Viability

In order to evaluate the cell proliferation, ARPE-19 cells were plated in 96-well plates (2 × 10^4^ cells/well) and cultured for 24, 72, or 96 hours in the presence or absence of 10 nmol/L GLP-1. Viable cells were identified using the Cell Titer 96 Aqueous One Solution Cell Proliferation Assay (Promega, Milan, Italy), accordingly to the manufacturer's instructions, as previously described [23].

### 2.3. Reverse Transcriptase-Polymerase Chain Reaction (RT-PCR)

Total RNA was extracted from ARPE-19 cells cultured with control medium alone with RNeasy kit (QIAGEN s.r.l., Milan, Italy) according to manufacturer's instruction. The RNA concentration was determined spectrophotometrically. One microgram of RNA was reverse-transcripted to cDNA using GoScript Reverse Transcription System (PROMEGA ITALIA, Milan, Italy) and then amplified by PCR. The set of primers for human GLP1R was purchased from QIAGEN S.p.A., Milan, Italy, and the resultant PCR product was 98 bp. Glyceraldehyde-3-phosphate dehydrogenase (GAPDH) was used as internal control. Primers for GAPDH were designed according to their mRNA sequences from the GenBank (sense: 5′-TGA AGG TCG GAG TCA ACG GAT TTG GT-3′; antisense: 5′-CAT GTG GGC CAT GAG GTC CAC CAC-3′). The resultant PCR product was 558 bp. The cDNA was amplified using the PCR Master Mix (PROMEGA ITALIA, Milan, Italy), each cycle consisting of 30 s at 94°C, 60 s at 62°C for amplifying GLP1R and GAPDH, with a final extension step of 10 min at 72°C. All the samples were amplified in a linear amplification range established using a serial cDNA dilution and varying the number of cycles (37 cycles for GAPDH and GLP1R). PCR products were electrophoresed onto a 1.5% agarose gel containing ethidium bromide and visualized under UV light. 

### 2.4. RNA Silencing

For the GLP-1R siRNA experiment a pool of 4 prevalidated siRNA designed for human GLP-1R were used (Dharmacon Accell si RNA reagents, Thermo Scientific, Milan, Italy). ARPE-19 cells were seeded in 12-well plates in culture medium without antibiotics and grown overnight to reach 60–80% confluence. The day after, Accell delivery mix (Dharmacon Accell si RNA reagents, Thermo Scientific, Milan, Italy) containing siRNA complexes was prepared according to manufacturer's instruction. Cells were transfected with GLP-1R siRNA or control siRNA (which correspond to a nontargeting 23-nucleotide siRNA designed as negative control). Transfection mixtures were left on the cells for 72 h, and then GLP-1R protein level was tested by immunoblotting. 

### 2.5. Immunoblotting Analysis

After treatments, ARPE-19 cells were lysed in RIPA buffer (50 mmol/L Tris HCl pH 7.5, 150 mmol/L NaCl, 1% NP40, 0.1% SDS, supplemented with protease and phosphatase inhibitor cocktails), and protein concentration was determined using the BCA protein assay Kit. Total cell lysate (30 micrograms) was separated on a SDS-PAGE and transferred onto nitrocellulose. Lysates from the pancreatic insulin secreting cell line HIT-T15 were used as positive control. Filters were blocked in 5% BSA and incubated overnight at 4°C with primary specific antibodies anti-GLP1R, or *β*-Actin (both from Santa Cruz Biotechnology, Inc. Santa Cruz, CA, USA). Secondary specific horseradish-peroxidase linked antibodies were added for 1 h at room temperature. Bound antibodies were detected using an enhanced chemiluminescence lighting system (Luminata Classico, Millipore, Billerica, MA, USA), according to manufacturer's instruction. Bands of interest were quantified by densitometry using the NIH program ImageJ. To verify equal loading of the proteins, membranes were stripped again, reblocked, and reprobed to detect *β*-actin.

To evaluate intracellular signalling pathways potentially activated by GLP-1, cells were cultured in serum-free medium for 24 hours and in the absence or presence of 10 nmol/L GLP-1 for different time points (up to 40 minutes). Cells were then lysed and total protein extract were used for western blot analysis. In parallel experiments, cells were prestimulated with pharmacological kinase inhibitors (10 *μ*mol/l U0126, for mitogen-activated protein kinase [MEK]1/2; 50 *μ*mol/l LY294002, for Phosphatidylinositol-3kinase [PI3K]) for 10 minutes; or 0.25 *μ*mol/l Tyrphostin AG 1478 for epidermal growth factor receptor (EGFR) for 30 minutes. Then, cells were stimulated in the presence or absence of 10 nmol/L GLP-1 for 10 minutes. After incubation, cells were lysed, and lysates were separated by SDS-PAGE and immunoblotted with specific antibodies anti-phospho-p44/42 MAPK thr202/tyr204 (ERK1/2) and phospho protein kinase B Ser 473 (PKB) (Cell Signaling Technology, Beverly, MA, USA). Membrane-were stripped and reprobed respectively with anti-ERK1/2 or PKB antibody (Cell Signaling Technology, Beverly, MA, USA) to normalize the blots for total protein levels.

### 2.6. Vascular Endothelial Cell Growth-Activated (VEGF-A) Secretion

ARPE-19 cells were cultured for 24 hours in the presence or absence of 10 nmol/L GLP-1. To quantify VEGF-A secretion, the conditioned media were collected and stored at −80°C until the assay was performed. Cells were then washed twice with PBS and lysed in RIPA buffer, and lysates were stored at −80°C. The lysate protein content was determined by the BCA Protein Assay Kit (Pierce, Rockford, MD, USA) according to the manufacturer's instructions. VEGF-A secretion was assessed by ELISA (Bender MedSystem, Vienna, Austria). VEGF-A concentration was calculated from standards curve and normalized to total protein concentration of the respective lysate.

### 2.7. Statistical Analysis

The results are representative of at least 3 experiments. All analyses were carried out with the GraphPad Prism 4.0 software (GraphPad Software, San Diego, CA, USA). Data were expressed as the mean ± SE and then analysed using Student's *t*-test. *P* value <0.05 was considered as statistically significant.

## 3. Results

### 3.1. GLP-1R is Expressed on ARPE-19 Cells

**Figure 1:**
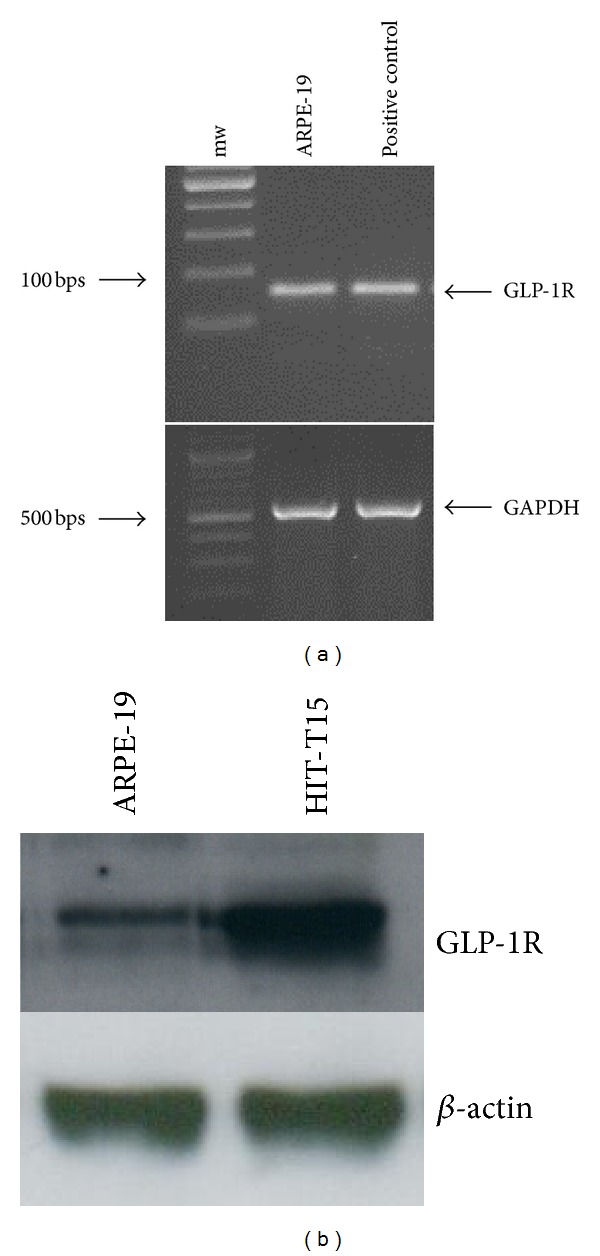
GLP-1R is expressed in ARPE-19 cells. (a) RT-PCR showing GLP-1R mRNA expression in human ARPE-19 cells cultured in standard medium. The expected RT-PCR product size is 98 bp. Glyceraldehyde 3-phosphate dehydrogenase (GAPDH) was utilized as an external control. Representative agarose gel of three different experiments. (b). Representative western blot analysis of human GLP-1R protein levels (*∼*65 kD) showing that GLP-1R protein is expressed in ARPE-19 cells. Lysates from the pancreatic insulin secreting cell line HIT-T15 were used as a positive control. Representative western blot analysis of three different experiments is shown.

The expression of GLP-1R on ARPE-19 cells was demonstrated by RT-PCR and western blot analysis. A band of about 100 bps was amplified through RT-PCR confirming mRNA expression of GLP-1R in ARPE-19 cells (Figure 1(a)). To verify transduction of mRNA into protein, western blot analysis with specific antibody anti-GLP1R was performed. The immunoblots revealed a well-detectable band between 60 and 70 kDaltons in the cytosolic protein extracts from ARPE-19 cells that was similar to the band shown in the positive control (HIT-T15 protein lysate) (Figure 1(b)).

### 3.2. GLP-1 Activates Defined Intracellular Pathways via Its Transmembrane Receptor GLP-1 on ARPE-19 Cells

**Figure 2:**
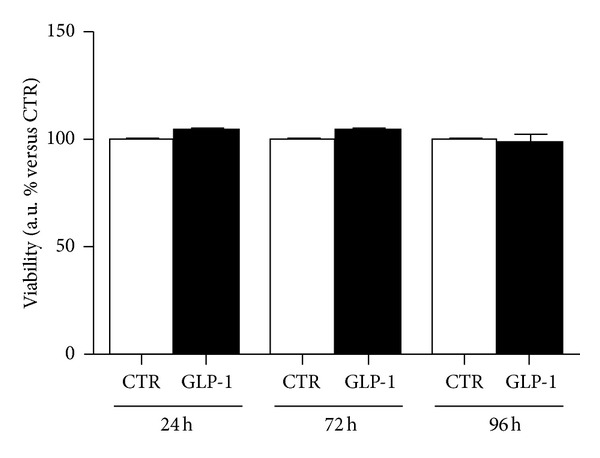
GLP-1 does not affect ARPE-19 cell viability. Cells were cultured for 24, 72, or 96 hours in the absence (CTR, white bars) or presence of 10 nmol/L GLP-1 (GPL-1, black bars). Cell proliferation rate was determined by a colorimetric method based on the formazan product of the tetrazolium compound MTS. Results showed the percentage of absorbance compared to CTR. Data were expressed as the mean ± SE of 3 independent experiments.

We first investigated whether stimulation with GLP-1 might affect ARPE-19 cell viability during *in vitro* protocol. Cells were cultured in the presence or absence of 10 nmol/L GLP-1 for 24, 72, or 96 hours. No difference in the ARPE-19 cell proliferation rate was shown between standard medium and GLP-1 at any time point investigated (Figure 2).

**Figure 3:**
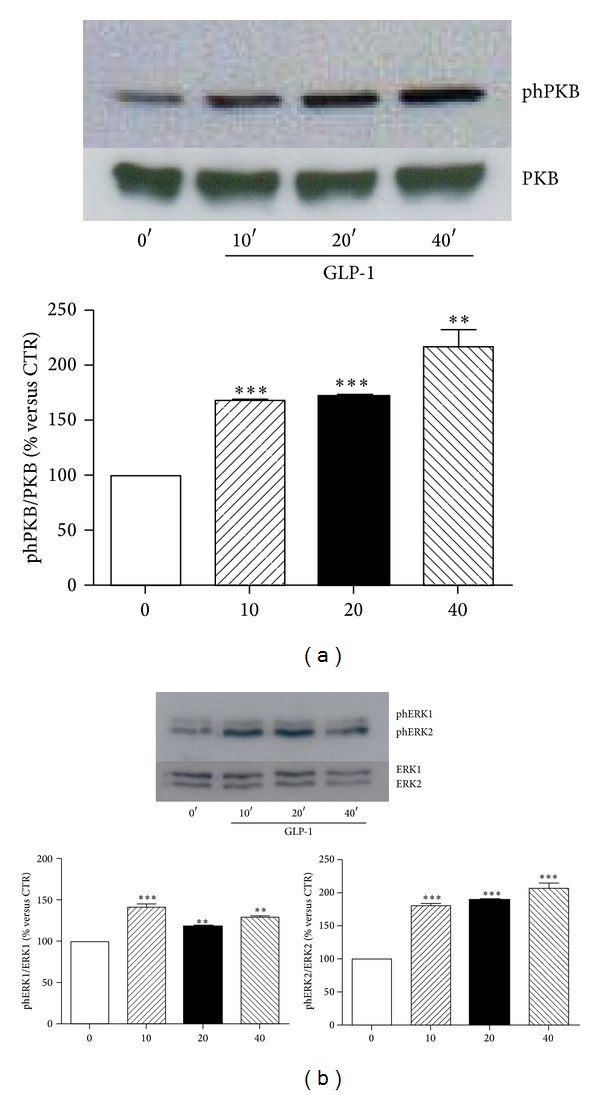
GLP-1 phosphorylates the intracellular signalling proteins PKB and ERK1/2. ARPE-19 cells were cultured in serum-free medium for 24 hours and stimulated with control medium (0′) or for 10, 20, and 40 minutes with 10 nmol/L GLP-1. Then, cells were then lysed and tested for phosphorylation and total protein of PKB (a) and ERK1/2 (b). Upper panel: representative western blot analysis of three different experiments is shown. Lower panel: densitometries of western blot bands. Data were expressed as mean ± SE of fold induction relative to total protein (*n* = 3). **P < 0.01 and ***P < 0.001 versus time 0′.

**Figure 4:**
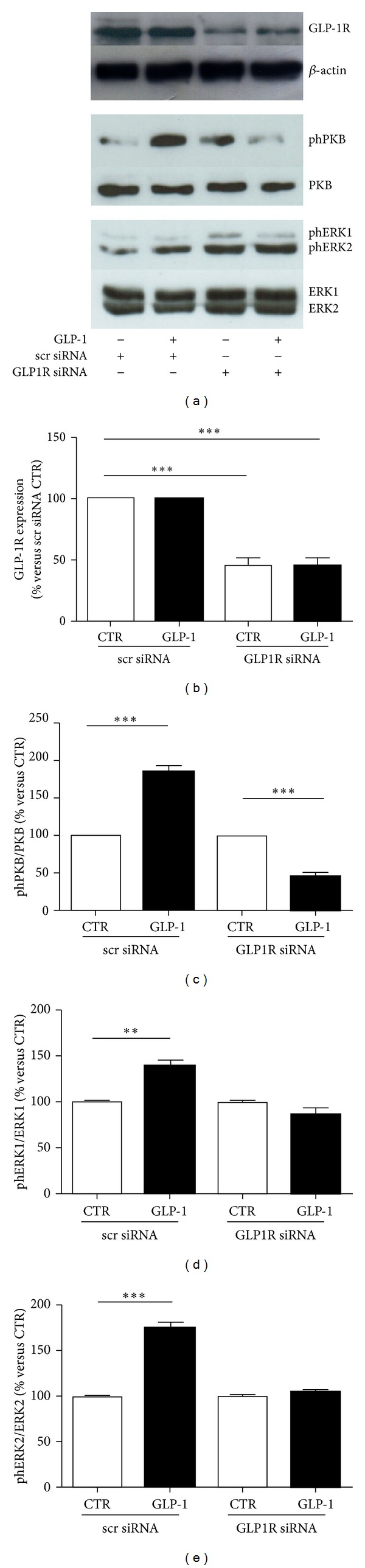
GLP-1R silencing abrogates GLP-1-induced signalling pathway activation. ARPE-19 cells were transfected with scrambled (scr) siRNA control or GLP-1R siRNA and treated with 10 nmol/L GLP-1 for 10 minutes. Cells were then lysed and tested with antibody anti-GLP-1R, anti-phPKB, or anti-phERK1/2. Membranes were stripped and reprobed, respectively, with anti-*β*actin, anti-PKB, or antiERK1/2-antibodies. (a) Representative western blot analyses of three different experiments are shown. (b) Quantification of densitometries of western blot bands of GLP-1R. Data were expressed as mean ± SE of fold induction relative to *β*-actin (*n* = 3). ***P < 0.001 versus scr siRNA CTR. (c) Quantification of densitometries of western blot bands of phPKB. Data were expressed as mean ± SE of fold induction relative to total PKB (*n* = 3). ***P < 0.001 versus respective CTR. (d) Quantification of densitometries of western blot bands of phERK1. Data were expressed as mean ± SE of fold induction relative to total ERK1 (*n* = 3). **P < 0.01 versus CTR. (e) Quantification of densitometries of western blot bands of phERK2. Data were expressed as mean ± SE of fold induction relative to total ERK2 (*n* = 3). ***P < 0.001 versus CTR.

We then investigated the potential function of GLP-1R in ARPE-19 cells. We focused on the downstream activation of postreceptor intracellular signalling pathways triggered by the binding of GLP-1 to its cognate GLP-1R. The incubation with GLP-1 increased the phosphorylation levels of ERK1/2 and PKB as compared to the control cells already after 10 minutes (Figures 3(a) and 3(b)). These effects on ERK1/2 and PKB phosphorylation remained significantly increased as compared to control medium at even 40 minutes of incubation (Figures 3(a) and 3(b)). To demonstrate that GLP-1 activated intracellular kinases via its cognate receptor, we downregulated GLP1R expression through siRNA. This method significantly reduced GLP1R protein levels as compared to scrambled siRNA control (Figures 4(a) and 4(b)). In particular, transfection resulted in more than 60% knockdown of GLP-1R (Figure 4(b)). In GLP1R silenced cells, treatment with GLP-1 failed to phosphorylate PKB and ERK1/2 (Figures 4(a), 4(c)–4(e)). On the other hand, in scrambles siRNA controls, GLP-1-induced PKB, and ERK1/2 phosphorylation were maintained (Figures 4(a), 4(c)–4(e)).

**Figure 5:**
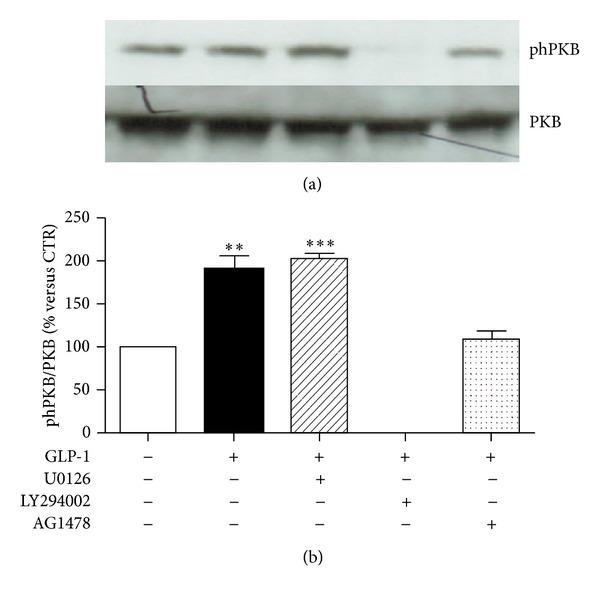
Pharmacological inhibition of PI3K abrogates GLP-1-induced activation of PKB. ARPE-19 cells were cultured in serum-free medium for 24 hours and stimulated for 10 minutes with 10 nmol/L GLP-1 in the presence or absence of MEK1/2 inhibitor U0126 (10 *μ*mol/l) or PI3K inhibitor LY294002 (50 *μ*mol/l), or EGFR inhibitor AG 1478 (0.25 *μ*mol/l). (a) Representative western blot analysis of PKB phosphorylation (phPKB) and total protein is shown. (b) Quantification of densitometries of western blot bands. Data were expressed as mean ± SE of fold induction relative to total PKB (*n* = 3). **P < 0.01; ***P < 0.001 versus cells stimulated with control medium alone.

**Figure 6:**
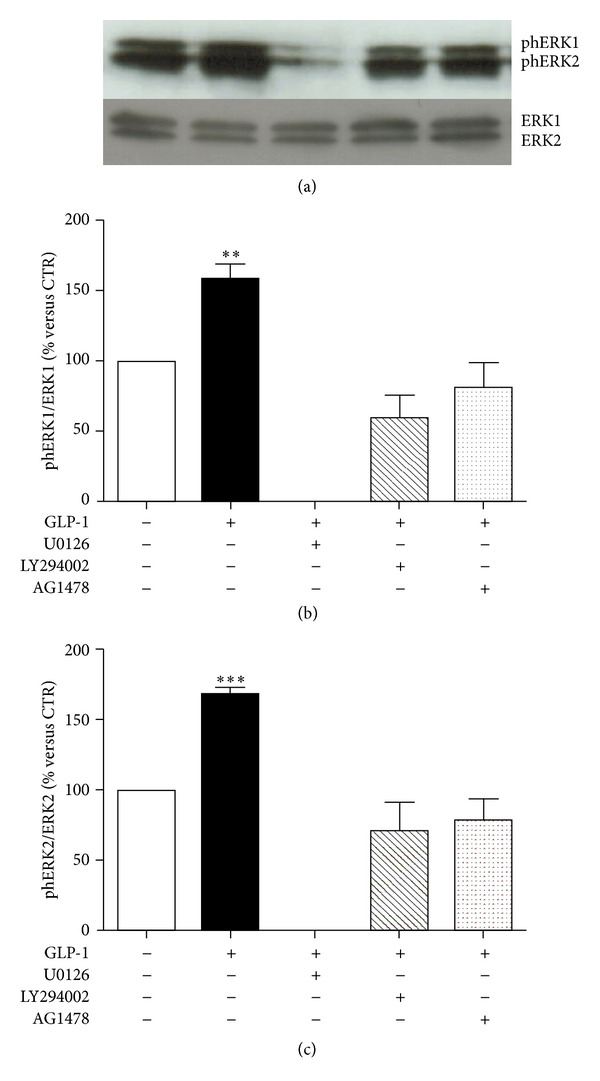
Pharmacological inhibition of PI3K blocked GLP-1-induced activation of ERK1/2. ARPE-19 cells were cultured in serum-free medium for 24 hours and stimulated for 10 minutes with 10 nmol/L GLP-1 in the presence or absence of MEK1/2 inhibitor U0126 (10 *μ*mol/l) or PI3K inhibitor LY294002 (50 *μ*mol/l), or EGFR inhibitor AG 1478 (0.25 *μ*mol/l). (a) Representative western blot analysis of ERK 1/2 phosphorylation (phERK1/2) and total protein is shown. (b) Quantification of densitometries of western blot bands of phERK1. Data were expressed as mean ± SE of fold induction relative to total ERK1 (*n* = 3). **P < 0.01 versus cells stimulated with control medium alone. (c) Quantification of densitometries of western blot bands of phERK2. Data were expressed as mean ± SE of fold induction relative to total ERK2 (*n* = 3). ***P < 0.001 versus cells stimulated with control medium alone.

Then, potential connections between PKB and ERK1/2 phosphorylation pathways were investigated. As expected (since PI3K is known to be a direct upstream activator of PKB in several cellular models) [24], the pharmacological inhibition of PI3K with LY294002 abrogated GLP-1-induced phosphorylation of PKB (Figures 5(a) and 5(b)). No effect on GLP-1-mediated phosphorylation of PKB was observed when cells were preincubated with the selective MEK1/2 inhibitor U0126 (Figures 5(a) and 5(b)). On the other hand, GLP-1-induced ERK1/2 phosphorylation was inhibited by preincubation with the selective inhibitor of MEK1/2 (the upstream activator of ERK1/2) (Figures 6(a)–6(c)) and with PI3K inhibitor LY294002 (Figures 6(a)–6(c)), suggesting that PI3K activation is also required for the GLP-1-induced activation of ERK1/2. Since it has been reported in other models that GLP-1R can activate PI3K through the transactivation of the epidermal growth factor receptor (EGFR) [25, 26], we also preincubated cells with AG1478 (a selective tyrosine kinase inhibitor of EGFR). Pretreatment with AG1478 was associated with inhibition of downstream phosphorylation of ERK1/2 and PKB in the presence of GLP-1 (Figures 5 and 6).

### 3.3. GLP-1 Does Not Affect Spontaneous Release of VEGF-A on ARPE-19 Cells

**Figure 7:**
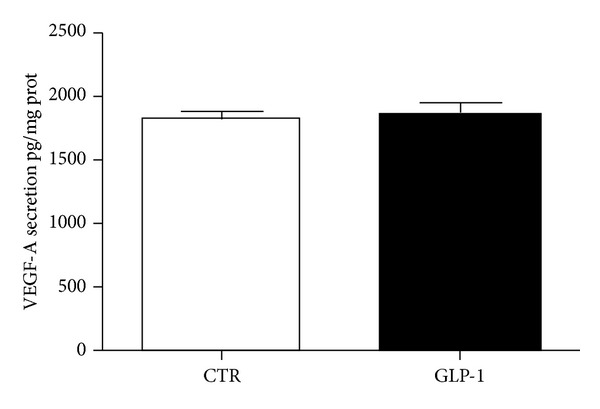
GLP-1 does not affect secretion of VEGF-A. ARPE-19 cells were cultured for 24 h in in the presence or absence of 10 nmol/L GLP-1. The conditioned media were collected, and VEGF-A levels in supernatants were assessed by ELISA. VEGF-A concentration was calculated from standards curve and normalized to total protein concentration of the respective lysates. The results are representative of three independent experiments (mean ± SE).

RPE has be shown as a primary source of the controversial mediator VEGF-A within the eye [27, 28]. Considering that treatments with GLP-1 receptor agonists were shown to improve retinal diabetic complications in animal models [14, 15], we investigated if this molecule might increase the spontaneous release of VEGF-A from ARPE-19 cells. No significant difference between incubation with GLP-1 and standard medium was shown (Figure 7).

## 4. Discussion

In this study, we showed that a GLP-1R is expressed in RPE cells. An emerging clinical interest in the management of type 2 diabetes has been shown for GLP-1. This hormone protects from diabetes and its complications improving both beta-cell pathophysiology and potentially within the peripheral organs (including the eye and kidney) targets of diabetic complications [12]. The demonstration of GLP-1R in RPE cells might suggest a novel protective role for GLP-1 also in a very common complication of DM, such as DR. This diabetic alteration of the retina is now considered as a neurodegenerative disease of the eye, with breakdown of the blood-retina barrier and loss of neuronal cells [16–18]. Recent studies have demonstrated the existence of GLP-1 receptors in the retinal nerve layer and that exenatide (a stable long-acting analogous of GLP-1) can prevent the loss of cells and maintain the normal retinal thickness in diabetic rats [14, 15]. Previous immunohistochemical studies showed that GLP-1R expression was mainly in the inner retina [14]. Here, we showed that GLP-1R is expressed also in the retinal pigment epithelium, that constitutes the outer blood-retinal barrier and therefore is essential for the integrity of the retina [29]. 

Another main result of this study is represented by a demonstration that GLP-1R is capable of transducing GLP-1-mediated signal within ARPE-19 cells. It has been reported that binding of GLP-1 to its cognate receptor resulted in the activation of different signalling pathways within various cell subsets [9–11] (mainly pancreatic beta-cells). In particular, GLP-1-triggered activation of PI3K was shown to be regulated by multiple integrated pathways, exerting different beta-cell functions, such as survival, metabolism, and channel regulation [21, 30–32]. GLP-1-mediated activation of PI3K resulted in the direct downstream phosphorylation of the serine/threonine kinase PKB, pivotal for beta-cell proliferation and survival [33, 34]. Similarly to PKB, the phosphorylation of ERK1/2 in response to GLP-1 treatment has been demonstrated in several insulinoma cell lines and in human islets [11, 35, 36]. In this study, we showed that GLP-1 via its cognate receptor GLP-1R significantly increased both downstream PKB and ERK1/2 phosphorylation in ARPE-19 cells. Importantly, the phosphorylation of ERK1/2 and PKB was detected after 10 min of exposure to GLP-1 and remained elevated even at 40 min. To further identify the potential interconnections between GLP-1R and these downstream intracellular pathways, we pretreated cells with selective inhibitors of some kinases that have been shown to directly activate ERK1/2 and PKB, respectively, an in other cell types [37, 38]. The results demonstrated that inhibition of PI3K completely abrogates GLP-1-induced phosphorylation of PKB and ERK1/2, suggesting that activation of PI3K was upstream to both kinases. On the other hand, the inhibition of MEK1/2 did not affect PKB, but only ERK1/2 (as expected), indicating that GLP-1 might trigger via its receptor GLP-1R PI3K-dependent intracellular pathways. Furthermore, accordingly with previous reports in other cell lines [25, 26], the concomitant inhibition of EGFR prevented the phosphorylation of ERK1/2 and PKB induced by GLP-1, suggesting that GLP-1R signalling may involve the transactivation of EGFR. Considering that the activation of these pathways by GLP-1 was often associated with an increased survival of injured cells [25, 26], in our study we did not observe difference in cell proliferation, suggesting that GLP-1 does not increase proliferation rate in healthy RPE cells. Finally, our finding that GLP-1 failed to increase the protective spontaneous VEGF-A secretion further supports the hypothesis that GLP-1 does not alter physiological function of ARPE-19 cells. 

This study has some limitations: first of all our experiments were carried out exclusively *in vitro* using a human cell line. This aspect dramatically weakened the relevance of our results. However, this study might represent the first demonstration and rationale for additional studies investigating a direct role of GLP-1/GLP-1R axis to prevent DR. A second limitation is represented by the limited number of retinal cell function investigated. The aim of this study is to demonstrated the presence of a functional GLP-1R on ARPE-19 cells. Thus, our results are preliminary to the exploration of a different potential GLP-1-mediated activity on retinal cells. 

## 5. Conclusions

In conclusion, our data showed that GLP-1R is expressed on ARPE-19 cells and that this receptor is directly activated by GLP-1. The underlying signalling pathways involve the PI3K- and EGFR-mediated activation of downstream PKB and ERK1/2. Knowledge that RPE cells may be responsiveness to GLP-1 suggested that GLP-1/GLP-1R axis might potentially contribute to prevent RPE cell dysfunction and, consequently, DR. These results represent a novel evidence for an unexplored mechanism by which long-acting agonists of GLP-1R (clinically administered to diabetic patients) might directly protect from a relevant and common diabetic complication such as DR.
